# Can only histological evaluation determine the allocation of ECD kidneys?

**DOI:** 10.1186/1471-2369-15-207

**Published:** 2014-12-23

**Authors:** Carlo Grifasi, Vincenzo D’Alessandro, Maria D’Armiento, Severo Campione, Alessandro Scotti, Luigi Pelosio, Andrea Renda

**Affiliations:** Department of Advanced Biomedical Sciences, Section of General Surgery, University “Federico II”, Naples, Italy; Department of Advanced Biomedical Sciences, Section of Anatomic Pathology, University “Federico II”, Naples, Italy; Interdepartmental Center for Kidney Transplantation, University “Federico II”, Naples, Italy; Dipartimento di Scienze Biomediche Avanzate, Università “Federico II”, via Pansini 5, 80131 Napoli, Italy

**Keywords:** Donor organ shortage, Expanded criteria donors, Dual kidney transplant, Pre-transplant biopsy

## Abstract

**Introduction:**

There is a recent debate on the "transplantability" of ECD (Expanded Criteria Donors) kidneys and the selection criteria used to allocate them to single or double transplantation. Remuzzi et al. have defined a protocol incorporating pre-transplant donor biopsy to guide the use of older donor organs. They allocated organs as single or double transplants on the basis of histological findings. We aim to show the pros and cons of the only histological evaluation in the allocation of ECD kidneys, to compare the different experiences in United States and Europe and thus to discuss whether this tool should be used alone or included in a comprehensive clinical and histopathological evaluation.

**Discussion:**

In the United States many Authors stated that the biopsy actually increases the percentage of kidney discarded and they raised questions about the importance of the biopsy in evaluating ECD kidneys for transplantation. On the other hand, the experiences of the majority of european transplant centers showed that allocating kidneys as single or dual transplant based on biopsy findings may achieve good graft and patient outcomes.

Moreover, the experience of some centers as ours showed that kidneys allocated as DKT (Dual Kidney Transplant) on the basis of Remuzzi’s score could have been suitable for single transplantation suggesting the need of an adjustment of the Remuzzi Score System. Many Authors, who are in favor of histological evaluation, actually believe that a comprehensive clinical and histopathological assessment before transplantation remains necessary.

**Summary:**

We lack precise national- or international-based selection criteria to guide clinicians. An adjustment of the Remuzzi Score System could be taken into consideration such as narrowing the indication for DKT from those ECD kidneys with higher scores and including the histological evaluation in a multifactor score.

## Introduction

The disparity between supply of deceased donors and demand has increased in the last decades and has led to the increased use of organs from marginal donors, mostly in form of expanded criteria donor (ECD) kidneys [1–7]. ECD is defined as all deceased donors ≥ 60 years of age or donor who were 50-59 years of age and had two of the following: donor history of hypertension; donor death due to cerebrovascular accident/stroke; or terminal serum creatinine value greater than 1,5 mg/dl.

Because of the decreased chance of survival of renal grafts from ECDs, the transplantation of two ECD kidneys into a single recipient (dual kidney transplantation[DKT]) has been introduced. It is considered when a single transplant exposes the recipient to the drawbacks of a limited nephron mass supply. This strategy has provided good outcomes in the experience of some transplant center [8–19].

To solve the problem of maximizing the use of ECD kidneys and choosing which ECD kidneys should be used for single or double use, it has become important to develop tools that permit an accurate evaluation of their "transplantability". Remuzzi et al. have defined a protocol incorporating pre-transplant donor biopsy to guide the use of older donor organs. They allocated organs as single or double transplants on the basis of histological findings. They reported excellent outcomes of the Dual Kidney Transplant Group at 5 years of follow-up [20]. However, other protocols to allocate organs have been proposed, that consider glomerular filtration rate (GFR) alone, or GFR in combination with clinical and histological parameters, so that there is not yet a standardized score to address ECD allocation.

We aim to show in this review the pros and cons of the only histological evaluation in the allocation of ECD kidneys, to compare the different experiences in United States (US) and Europe and thus to discuss whether this tool should be used alone or included in a comprehensive clinical and histopathological evaluation.

## Discussion

### "Biopsy results" as a major reason for discard of ECD kidneys

In European countries, lack of elderly recipients on the waiting list as well as macroscopic or microscopic kidney abnormalities were the main causes for organ discard. On the other hand, biopsy findings and machine perfusion have revealed major reasons to discard ECD in the United States.

US transplant centers continue to discard an high percentage of deceased donor kidneys that have been procured for transplantation. Between October 1999 and June 2005, more than 9000 donated kidneys were determined to be unsuitable for transplantation, including 41% (5139 of 12536) of the expanded criteria donor (ECD) kidneys procured, according to an analysis from the Scientific Registry of Transplant Recipients in this issue [21]. Sung et al. have also identified ‘biopsy results’ as a major reason for discard of ECD kidneys and use of pulsatile perfusion for kidney preservation as a mitigating factor.

Moreover, the Authors considered that there may be important selection biases at work that cannot be detected in Registry analyses and that the degree of glomerulosclerosis is the only biopsy result that can be evaluated from the forms that are submitted. Glomerulosclerosis does not appear to be accurate histologic indicator risk of subsequent renal function [22]. Probably the different impact of "biopsy results" on the discard rate of ECD kidneys in US and Europe might be linked to the variability in interpretation of kidney biopsies; for instance, many studies successfully linked arterial hyalinosis, fibrous intimal thickening, tubular atrophy and interstitial fibrosis to clinical outcomes. In the future, more uniform and inclusive biopsy report system (vessels, glomeruli, tubules and interstitium) will be necessary to utilize histopathology more efficiently in the allocation system.

To develop a tool to reduce the discard rate of marginal kidneys in US, the United Network for Organ Sharing (UNOS) Kidney Transplantation Committee has approved a new allocation system on June 25, 2013. This new system uses the Kidney Donor Risk Index (KDRI) to steamline allocation of kidneys which otherwise would have been discarded. The KDRI combines a variety of donor factors to summarize the risk of graft failure after kidney transplant into a single number. The following donor characteristics are used to calculate the KDRI: age, height, weight, ethnicity, history of hypertension, history of diabetes, cause of death, serum creatinine, Hepatitis C Virus (HCV) status, Donation after Circulatory Death (DCD) status. The KDPI (Kidney Donor Profile Index) is a remapping of the KDRI onto a cumulative percentage scale, such that a donor with a KDPI of 80% has higher expected risk of graft failure than 80% of all kidney donors recovered last year. Clinical applicability of a continuous variable has limitations in a binary decision-making process like acceptance or rejection of a kidney (if there is not a cutoff). Thus one should have great caution in utilizing KDPI to predict a specific patient's suitability for that organ or for discriminating kidneys that are suitable for transplantation [23, 24].

With the goal to find a tool for identification of ECD kidneys that can be accepted for tranplantation, Gandolfini et al. [25] analyzed the contribution of donor biopsies in the acceptance or rejection of marginal kidneys. The majority of the kidneys in this study qualified to be extended criteria donor (ECD) kidneys, in this sense it may be considered as confirmatory study of a previous study by Remuzzi et al. Similar to the previous study by Remuzzi et al, the use of pretransplant biopsies increased utilization of marginal kidneys in this cohort of patients. The Authors also characterized these kidneys with the Kidney Donor Profile Index (KDPI) and used this for further analysis. They found that kidneys with the highest KDPI might have superior outcomes with a lower Remuzzi score than those with a higher score. The results of the current study are far from definitive in the controversy over the utility of pretransplant biopsies, but offer added evidence that perhaps in marginal/ECD/high KDPI kidneys biopsy data may have some incremental utility. The study by Gandolfini et al. gives an opportunity to reflect on the role of KDPI scoring system which is not a decision tool but only a score for better characterizing potential organ donors.

In order to reduce discard rate of marginal kidneys a potential option is to allocate them as a DKT. In Europe DKT proved to be a successful strategy for compensating the declining number of cadaver donor kidney transplants. On the other hand DKT is not a part of the allocation system in US and comprises less than 2% of the deceased donor transplants performed. The criteria proposed by UNOS recommend consideration of DKT if any two of the following criteria exist: donor age greater than 60 years; estimated donor creatinine clearance less than 65 ml/min, rising serum creatinine (greater than 2,5 mg/dl) at time of retrieval; history of medical disease in donor (defined as either longstanding hypertension or diabetes mellitus); adverse donor kidney histology, which is defined as moderate to severe glomerulosclerosis (greater than 15% and less than 50%). Potential factors associated with low utilization of DKT in US are higher surgical risk, longer ischemic time and the uncertainty in determining which organs are suitable for single versus dual use.

### The need of an adjustment of the Remuzzi Score System

According to the prospective cohort study of the Dual Kidney Transplant Group in Italy, the pretransplant biopsy is an important tool to optimize the allocation and utilization of ECD kidneys.

Nevertheless the experience of some centers as ours showed that kidneys allocated as DKT on the basis of Remuzzi’s score could have been suitable for single transplantation. Cruzado et al. [26] demonstrated in their study that 5- and 10-year death-censored graft survivals were better in DKT than patients who had lost one of their grafts (the uninephrectomized (UNX) UNX group); however, graft survival among the UNX group may be considered acceptable, taking into account the donor shortage. Timsit et al. [27] also compared outcomes between a group of patients who underwent explantation of one of the grafts (the SINGLE group) and a group of patients who had two functional grafts (the DUAL group). One-year glomerular filtration rate was significantly lower in the SINGLE group (29.4 ml/min/1.73 m2 vs. 49.4 ml/min/1.73 m2 in the DUAL group, P < 0.05). Significantly, none of the nine recipients of the SINGLE group returned to dialysis with a mean follow-up of 34.1 months.

Other studies showed the need of an adjustment of the criteria proposed by Remuzzi et al. For instance, Fernandez-Lorente et al. [28] have evaluated long-term results of biopsy-guided allocation of kidneys in their old-for-old program. Interestingly, they found that in single kidney transplantation (SKT) long-term graft survival was similar for Remuzzi’ s score 4 and ≤3. Moreover, in DKT the score 4,5 and 6 had no influence on graft survival. These results suggested that only kidneys with scores 5 and 6 should be allocated for DKT. These findings are similar to those of a study of Losappio et al. [29] which showed that allocation of kidneys with a score of 4 to SKT provides an acceptable long-term graft survival. These results are against the original criteria proposed by Remuzzi, which stated that kidneys with a score between 4 – 6 should be used as DKT.

### Assessment of the "transplantability" of ECD kidneys: single criterion vs. multifactor score

There are several methods reported in literature to assess the quality of ECD kidneys and so to allocate them to single or dual transplant. Transplantation centers may make this decision based on a single criterion or on a comprehensive clinical and histological assessment. In 2006, Remuzzi et al. reported the long-term graft survival of 62 patients who received a transplant (single or dual) from donors over 60 years of age. In this study, marginal kidneys with no macroscopic abnormalities were allocated on the basis of the only histological evaluation of the grafts before transplantation: histological changes in the vessels, glomeruli, tubules, and connective tissue in biopsy specimens received a score from 0 (no changes) to 3 (severe changes). When both donor kidneys had a total score of 0 – 3, each kidney was used for a SKT. When the total score was 4 – 6, the two kidneys were transplanted in the same recipient (DKT). Kidneys with a total score ≥ 7 were not utilized. 3 years after transplantation, recipients of SKT and DKT organs from donors over 60 years of age that had undergone preimplantation biopsy had a mean graft survival rate > 90%, which was similar to that observed in a matched cohort of recipients of SKT renal grafts from donors < 60 years of age. Moreover, the survival rate of grafts from donors > 60 years of age that had undergone preimplantation biopsy was significantly higher than that observed in a matched cohort of recipients of organs from donors > 60 years of age that had not undergone preimplantation biopsy (93% versus 72%). Other studies have tried to define the predictive value of pretransplant biopsy with opposed results. For instance, B. Ekser et al. [30, 31] use the North Italian Transplant Program (NITp) allocation criteria for older donors. Histological assessment forms part of the NITp allocation criteria. In their recent report, the Authors did not describe the detailed assessment of pretransplantation histopathology. However, they emphasized that the Remuzzi Score System was used in making the final decision on the allocation of the kidneys. They concluded that pretransplant kidney biopsy optimizes the utilization and allocation of kidneys from ECDs and may lead to optimal outcomes not only from donors >60 years but also from donors >70 years. But the experience of Ekser et al. is a single-center, nonrandomized experience on the utilization and value of pretransplant biopsy. On the other hand, Re et al [32] performed a retrospective study among a group of kidney transplant recipients to evaluate postransplant evolution with clinical (Deceased Donor Score [DDS]) and histopathological (Remuzzi [REM]) scores. Briefly, the DDS is based on 5 donor clinical variables: age, history and duration of hypertension, cerebrovascular disease as cause of death, final creatinine clearance, and number of HLA mismatches. These Authors showed a significant correlation of DDS with serum creatinine values over 1 and 2 years. REM did not show a significant association with any event.

However, other scores have been proposed to allocate organs, such as glomerular filtration rate (GFR) alone, or GFR in combination with clinical, epidemiological, or histological criteria, but it has not defined a standardized score to address ECD allocation yet [33–46]. Moreover, according to Goldberg et al. the donor-recipient size mismatch affects post-transplant outcomes and should be included in the assessment of the organs [47].

## Summary

The transplant community should develop strategies to maximize the yield of the existing donor pool. The use of kidneys from ECD may permit more transplantations. For ECD kidneys unsuitable for single use, DKT may be possible. In many centers kidneys from ECD are histologically evaluated before implantation to decide the allocation for single or double use, but other scores have been proposed to allocate organs and there is not yet a standardized score to address ECD allocation. According to some studies, only kidneys with Remuzzi’s score of 5 and 6 should be used for DKT. Therefore, an adjustment of the Remuzzi Score System could be taken into consideration such as narrowing the indication for DKT from those ECD kidneys with higher scores and including the histological evaluation in a multifactor score.

## Authors’ information

CG, specializzando in Chirurgia Generale, Dipartimento di Scienze Biomediche Avanzate, Università Federico II, Naples, Italy

VD, dirigente medico, Dipartimento di Scienze Biomediche Avanzate, Università Federico II, Naples, Italy

MD, ricercatore di Anatomia Patologica, Dipartimento di Scienze Biomediche Avanzate, Università Federico II, Naples, Italy

SC, specializzando in Anatomia Patologica, Dipartimento di Scienze Biomediche Avanzate, Università Federico II, Naples, Italy

AS, dirigente medico, Dipartimento di Scienze Biomediche Avanzate, Università Federico II, Naples, Italy

LP, dirigente medico, Dipartimento di Scienze Biomediche Avanzate, Università Federico II, Naples, Italy

AR, professore ordinario di Chirurgia Generale, Dipartimento di Scienze Biomediche Avanzate, Università Federico II, Naples, Italy

## Abbreviations

SKT: Single kidney transplantation
DKT: Dual kidney transplantation
UNX: Uninephrectomized
ECD: Expanded criteria donor
DDS: Deceased donor score
UNOS: United Network for Organ Sharing
KDRI: Kidney Donor Risk Index
KDPI: Kidney Donor Profile Index.
